# Acquired TET2 mutation in one patient with familial platelet disorder with predisposition to AML led to the development of pre‐leukaemic clone resulting in T2‐ALL and AML‐M0

**DOI:** 10.1111/jcmm.13051

**Published:** 2016-12-20

**Authors:** Vladimir T. Manchev, Hind Bouzid, Iléana Antony‐Debré, Betty Leite, Guillaume Meurice, Nathalie Droin, Thomas Prebet, Régis T. Costello, William Vainchenker, Isabelle Plo, M'boyba Diop, Elizabeth Macintyre, Vahid Asnafi, Rémi Favier, Véronique Baccini, Hana Raslova

**Affiliations:** ^1^ INSERM UMR 1170Gustave RoussyUniversité Paris‐SaclayEquipe Labellisée par la Ligue Nationale Contre le CancerVillejuifFrance; ^2^ Université Paris DiderotParisFrance; ^3^ Gustave RoussyUniversité Paris‐SaclayGenomic Platform UMS AMMICAVillejuifFrance; ^4^ Gustave RoussyUniversité Paris‐SaclayBioinformatic Core Facility UMS AMMICAVillejuifFrance; ^5^ Faculté de MédecineAix‐Marseille UniversitéMarseilleFrance; ^6^ Département d'HématologieInstitut Paoli‐CalmettesMarseilleFrance; ^7^ Assistance Publique‐Hôpitaux de MarseilleHôpital de La ConceptionService d'Hématologie et Thérapie CellulaireFaculté de MédecineAix‐Marseille UniversitéINSERM UMR 1090 TAGCMarseilleFrance; ^8^ Hematology and INSERM U1151Institut Necker‐Enfants MaladesUniversité Sorbonne Paris CitéDescartes and Assistance Publique‐Hôpitaux de ParisParisFrance; ^9^ Assistance Publique‐Hôpitaux de ParisHôpital TrousseauService d'Hématologie biologiqueParisFrance; ^10^ Assistance Publique‐Hôpitaux de MarseilleHôpital NordLaboratoire d'HématologieFaculté de MédecineAix‐Marseille UniversitéINSERM UMR_S 1062MarseilleFrance

**Keywords:** FPD/AML, predisposition to leukaemia, RUNX1, TET2, AML‐M0, T2‐ALL

## Abstract

Familial platelet disorder with predisposition to acute myeloid leukaemia (FPD/AML) is characterized by germline *RUNX1* mutations, thrombocytopaenia, platelet dysfunction and a risk of developing acute myeloid and in rare cases lymphoid T leukaemia. Here, we focus on a case of a man with a familial history of *RUNX1* ^R174Q^ mutation who developed at the age of 42 years a T2‐ALL and, 2 years after remission, an AML‐M0. Both AML‐M0 and T2‐ALL blast populations demonstrated a loss of 1p36.32‐23 and 17q11.2 regions as well as other small deletions, clonal rearrangements of both TCRγ and TCRδ and a presence of 18 variants at a frequency of more than 40%. Additional variants were identified only in T2‐ALL or in AML‐M0 evoking the existence of a common original clone, which gave rise to subclonal populations. Next generation sequencing (NGS) performed on peripheral blood‐derived CD34^+^ cells 5 years prior to T2‐ALL development revealed only the missense *TET2* ^P1962T^ mutation at a frequency of 1%, which increases to more than 40% in fully transformed leukaemic T2‐ALL and AML‐M0 clones. This result suggests that *TET2* ^P1962T^ mutation in association with germline *RUNX1* ^R174Q^ mutation leads to amplification of a haematopoietic clone susceptible to acquire other transforming alterations.

## Introduction

Acquired genetic alterations in *RUNX1* are frequently associated with numerous myeloid malignancies, especially acute myeloid leukaemia (AML) (40% of FAB M0 immature AMLs 1) and more rarely with T cell acute lymphoblastic leukaemia (T‐ALL) (25% of early thymic immature T‐ALL (ETP‐ALL) 2).

Germline *RUNX1* mutations are found in FPD/AML characterized by thrombocytopaenia, and a 35% life‐time risk of developing myelodysplastic syndrome (MDS) and/or AML. 3 T‐ALL development has also been reported in rare cases 4, 5. In AML, contrary to T‐ALL, the leukaemic transformation is almost always associated with a somatic *RUNX1* mutation on the second allele 4, 6, 7. Acquired mutations in *CDC25C* and *GATA2* have also been identified at a high frequency but only in a Japanese cohort 4, 7, 8, 9, suggesting that environmental and/or ethnic factors may play an important role in leukaemia transformation. Somatic mutations in *FLT3*,* PHF6*,* KIT*,* KRAS*,* RAD21*,* BCOR*,* BCORL1*,* CBL*,* CEBPA*,* MPL*,* TP53*,* WT1*,* SRSF2*,* DNMT3A*,* TET2* and *ASXL1* were described in patients who developed AML/MDS and mutations in *PHF6*,* WT1*,* NOTCH1*,* FLT3* and *ASXL1* in patients with T‐ALL 4, 5, 7, 9.

## Materials and methods

### Patient samples

Biologic samples of one FPD/AML pedigree were collected between 2006 and 2013 after informed consent, in accordance with the Declaration of Helsinki. Genomic DNA was extracted from fibroblasts, CD34^+^ cells, peripheral blood mononuclear and bone marrow total cells using kit Qiagen (Les Ulis, France).

### CGH arrays

Comparative genomic hybridization (CGH) arrays were performed on human CGH 2x400K (G4448A) (Agilent, Les Ulis, France) by hybridization of sample *versus* normal‐matched commercial reference. CGH data and protocols have been submitted to ArrayExpress at the EBI with the accession number E‐MTAB‐4623.

### Exome capture and sequencing (v4+UTR, 70 Mb)

Library preparation, capture, sequencing and variant detection have been carried out by IntegraGen. Exons were captured from blood DNA using Agilent SureSelect Human All Exon v5–70 Mb kit and sequenced on IlluminaHiSEQ 2000 instrument as previously described 10. WES data and prodtocols have been submitted to ArrayExpress at the European Bioinformatics Institute (EBI) with the accession number E‐MTAB‐4679. Variants present in the internal control and in public databases at a frequency of >1% were excluded, and only non‐synonymous mutations predicted by PolyPhen2 to be probably or possibly deleterious were analysed.

### Targeted NGS

The mutated regions were amplified by PCR using primers listed in Table S1. PCR products were end‐repaired, extended with an ‘A’ base on the 3′ end, ligated with indexed paired‐end adaptors (NEXTflex, Bioo Scientific, Saint Marcel, France) using the Bravo Platform (Agilent) and amplified by PCR for four cycles. Amplicon libraries were sequenced in an IlluminaMiSeq flow cell using the onboard cluster method, as paired‐end sequencing (2 × 250 bp reads) (Illumina, San Diego, CA, USA).

## Results

We focus on a man with a familial history of *RUNX1* ^R174Q^ mutation, previously referenced as AII‐1 11, 12, who developed at the age of 42 years an EGIL (European Group of Immunological Characterization of Leukemia) T2‐ALL with a loss of the 1p36 and 17q12 regions and the *ASXL1* ^R693^* mutation in the leukaemic clone. The patient was pre‐treated with corticosteroids and then treated according to the French GRAALL2003 protocol based on a polychemotherapy 13. As 15 days later the blasts were still present in the bone marrow, a second induction course with idarubicin and cytarabine was performed. The patient achieved complete remission 5.

Two years later, he was admitted to the hospital for relapse with 87% blasts in peripheral blood. The clinical course was aggressive and the patient died 1 month after hospitalization. The blasts exhibited basophilic cytoplasm and a lack of azurophilic granules (Figure S1A) and were characterized by immature marker expression (CD34^+^, CD13^+^, HLA‐DR^+^, CD33^low^, CD7^+^ and CD56^low^) and absence of cytoplasmic and surface expression of CD3 and cyMPO (Figure S1B, C). This phenotype was initially described by Suzuki as myeloid/NK‐cell precursor leukaemia 14 and represents a distinct subtype of AML‐M0 15. In contrast to previously described cases, we detected low expression of TdT, CD10, CD19, cyCD79a and CD5 (Figure S1B, C).

To investigate whether initial (T2‐ALL) and relapsed (AML‐M0) leukaemic blasts originated from the same clone, we performed first aCGH on both blast populations. In both samples, aCGH revealed loss of 1p36.32‐23 and of 17q11.2 and other small deletions (Table S2). One additional deletion on chromosome 11 and five duplicated regions on chromosome 14 were found exclusively in T2‐ALL (Table S2). In the AML‐M0 blasts, the loss of the 1p36.32‐23 region was associated with two large amplifications at the same break point (1p36.33‐32 and 1p36.23‐1p31.2) surrounding the deleted region (Figure S2). Other additional deletions and duplications were identified (Table S2). These results suggested that T2‐ALL and AML‐M0 blasts originated from the same clone, but harboured also specific features acquired during disease progression. Furthermore, identical clonal rearrangements of TCRγ (Vγ9‐Jγ1.1) and TCRδ (Dδ2‐Jδ1 and Dδ2‐Jδ3) (Figure S3 and data not shown) were identified in both T2‐ALL and AML‐M0 blasts strongly supporting their clonal affiliation. Finally, analysis of WES revealed 34 variants in the T2‐ALL and 49 in the AML‐M0. Interestingly, 20 variants were common between these two cell populations (Table 1). Taking into consideration that T2‐ALL blasts represented about 58% of the sequenced DNA and AML‐M0 blasts 87%, 18 genes were found to be mutated in the original clone at a frequency of more than 40% (or about 100% for X‐linked genes) (Table 1), suggesting that they were all present as heterozygous mutations. The results were confirmed by NGS (Table S3). Alterations in genes frequently associated with myeloid and/or lymphoid malignancies, such as *PHF6*,* EZH2*,* ASXL1*,* JAK1*,* JAK3*,* TET2* and *NOTCH1*, were found. We also identified nine probably deleterious (*CXCR4, IRS4, HCFC1, CTNND2, NRF1, PTPN13, PPP2R2B, MSRB2* and *MYO1D*) and two possibly deleterious *de novo* variants (*EPHA10, PCNXL2*) in genes that were also found with a frequency of >40% but had not previously been causally linked to AML/MDS. A second heterozygous *NOTCH1* ^K1488N^ mutation occurred only in the T2‐ALL (Table 2), while the initial *NOTCH1* ^K1607delinsNAK^ mutation became homozygous in AML‐M0 (Table 1). PRB4 mutated with an approximate 40% frequency only in T2‐ALL could be a passenger mutation. In the AML‐M0, only one variant was present at 50% but absent in T2‐ALL, *SUDS3* ^R325H^ (Table 3). SUDS3 is a subunit of the histone deacetylase‐dependent SIN3A corepressor complex interacting with RUNX1 and its genetic alterations could lead to a deregulation of RUNX1 transcriptional program. No acquired *RUNX1* mutation was found in T2‐ALL and AML‐M0.

**Table 1:** Gene mutations common to both T2‐ALL and AML‐M0

Gene, chr	Protein	Variant frequency in T2‐ALL (%)	Variant frequency in AML‐M0 (%)
PHF6, chrX	S247Y	63	96
IRS4, chrX	G1154V	49	92
HCFC1, chrX	R1982H	55	89
CXCR4, chr2	V116Afs*55	36	80
EZH2, chr7	S669N	34	44
EPHA10, chr1	R268H	30	29†
CTNND2, chr5	R82*	28	48
NRF1, chr7	R206W	27	50
ASXL1, chr20	R693*	27	52
PTPN13, chr4	R1838Q	25	47
PPP2R2B, chr5	K363Q	25	51
JAK1, chr1	R879H	24	54
JAK3, chr19	R657Q	21	55
TET2, chr4	P1962T	23	47
MSRB2, chr10	T59I	23	42
PCNXL2, chr1	R1169W	21	52†
MYO1D, chr17	R560Q	21	48
**NOTCH1, chr9**	**K1607delinsNLQ**	**29**	**97**
OPRD1, chr1	I279N	18	36
RRP7A, chr22	F241del	11	20

*stop codon, ^†^possibly damaging, in bold: mutation present at heterozygous state in T2‐ALL and at homozygous state in AML‐M0.

**Table 2:** Genes mutated only in T2‐ALL

Gene, chr	Protein	Variant frequency in T2‐ALL (%)
NOTCH1‐bis, chr9	K1488N	20
PRB4, chr12	G164R	20
ITGAX, chr16	D810Y	19
AQP5, chr12	L74M	14†
GRIN3B, chr19	L824M	14
CUBN, chr10	A2914S	13
OBSCN, chr1	A8630E	12
SCARF2, chr22	R147S	12
DDX3X, chrX	G11W	12
PDZD2, chr5	L34I	11
MCM3, chr6	A461D	11
WNT3, chr17	R60S	11
ZIK1, chr19	A364delinsVLYF*	11
BCOR, chrX	L1203Sfs*33	11

*stop codon, ^†^possibly damaging, in bold: mutation present at heterozygous state in T2‐ALL and at homozygous state in AML‐M0.

**Table 3:** Genes mutated only in AML‐M0

Gene, chr	Protein	Variant frequency in AML‐M0 (%)
SUDS3, chr12	R325H	50
SAP130, chr2	G803‐G804insPTQN	36
GGT1, chr22	R107C	30
LDB2, chr4	K355N	27
ENPP2, chr8	V785M	26
ATP8B1, chr18	R941G, G940V	23, 23
LOC441155, chr6	A81T	22
PVRIG, chr7	C62F	19
MMEL1, chr1	P743H	18
TMEM181, chr6	E111*	18
RBFOX3, chr17	E263*	18
GCGR, chr17	E362*	18
CENPC, chr4	E302*	17
PHACTR2, chr6	G201C	17
MUC20, chr3	T447M	15
FAM71F2, chr7	P198H	15
WDR60, chr7	G1009C	15
CSH2, chr17	V27I	15
BAG6, chr6	P252T	14
BCLAF1, chr6	S38Yfs*38	14
MYO9B, chr19	A1659S	14
SLC17A7, chr19	R314S	14
WDR52, chr3	E1818*	12
SOX7, chr8	P209T	12
C19orf26, chr19	G349W	12
MXRA5, chrX	E937*	12
SOBP, chr6	D569Y	11
HSPA12B, chr20	R650S	11
SUV39H1, chrX	C41*	11

*stop codon.

**Figure 1:**
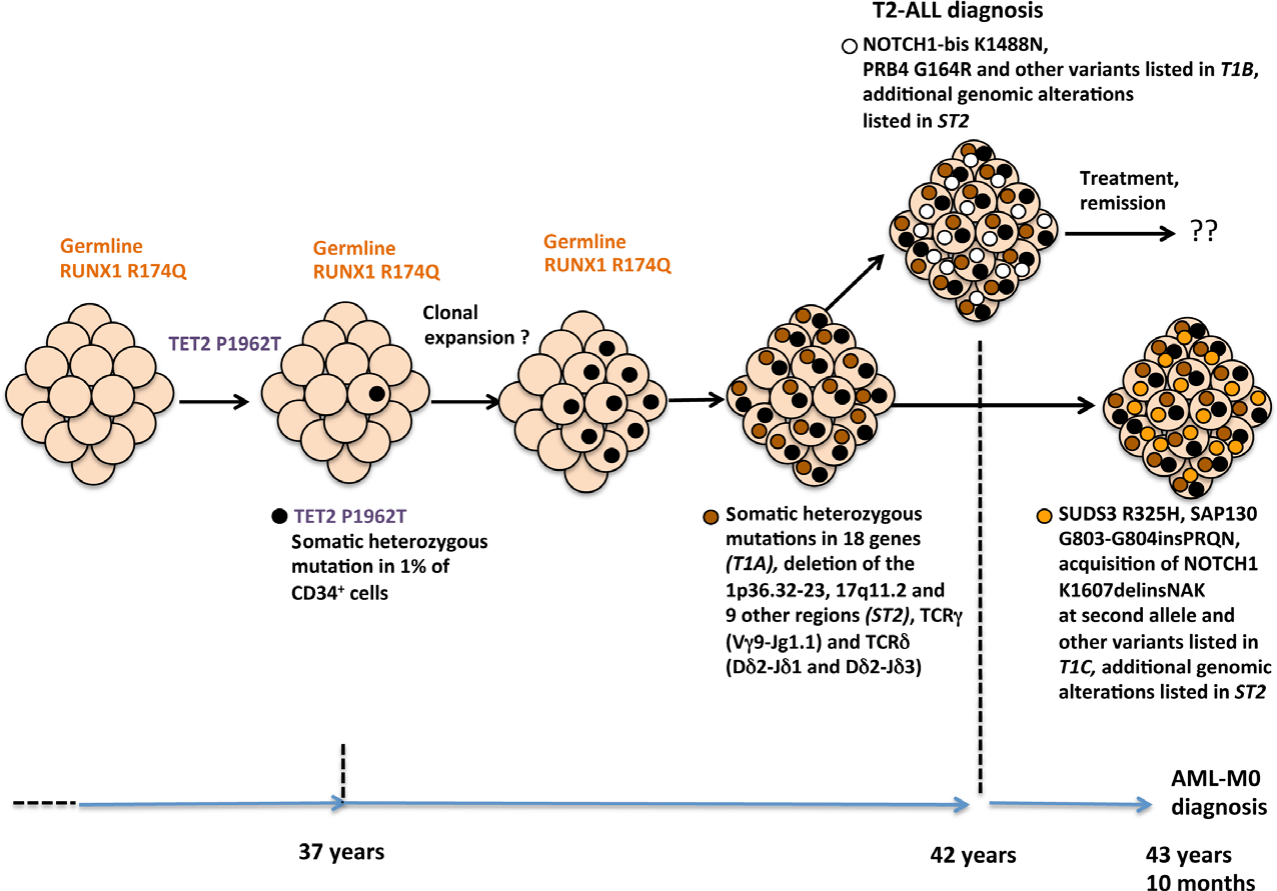
Clonal evolution in FPD/AML patient carrying germline *RUNX1* ^R174Q^ mutation. The clonal evolution with the different mutations occurring at various stages of the disease (thrombocytopaenia, T2‐ALL, AML‐M0) is represented. Frequency of the variants is listed in the Tables 1, 3. The other deletions and amplifications are listed in Table S2.

To examine whether some of the potentially deleterious variants accumulated before leukaemia development, we screened 20 variants found with a high penetrance in both T2‐ALL and AML‐M0 clones by NGS on CD34^+^ cells obtained from the patient's peripheral blood 5 years prior to T2‐ALL development. The only somatic mutation identified was a *TET2* ^P1962T^ at a frequency of 1% (Table S3) reaching more than 40% at leukaemic stages. These results suggest that a missense *TET2* ^P1962T^ mutation in association with germline *RUNX1* ^R174Q^ mutation led to amplification of an haematopoietic clone susceptible to acquire other transforming alterations (Fig. 1).

## Discussion

Here, we describe a unique case of one FPD/AML patient who developed first one T2‐ALL and less than 2 years later an AML‐M0. We show that these two leukaemic clones originate from the common subclone harbouring alterations in genes frequently mutated in both types of leukaemia. We cannot exclude that AML‐M0 was induced by the chemotherapy used for T2‐ALL treatment, as it involved anthracyclines and cyclophosphamide known to generate genomic instability. Interestingly, one of the variant *TET2* ^P1962T^ was present at a frequency of 1% in haematopoietic progenitors of this patient 5 years prior to T2‐ALL development. A missense *TET2* mutation at the same position (P1962L) has been described in AML 16 and T‐lymphoblastic lymphoma 17, and the role of TET2 in the clonal haematopoiesis preceding transformation in FPD/AML has been recently reported in another case of FPD/AML 18. However, this is the first report of a TET2^P1962T^ mutation leading to the amplification of a leukaemic clone.

Our results suggest the following model of transformation: the germline *RUNX1* mutation induces both alteration in haematopoiesis (more particularly increasing cycling) and a genetic instability 11. The acquisition of a somatic *TET2* mutation enhances self‐renewal of haematopoietic stem/progenitor cells 17, 19, 20, leading to a clonal haematopoiesis. This is not a clonal haematopoiesis of undetermined potential (CHIP) in this germline context, but a true pre‐leukaemic state responsible for the emergence of progressively divergent haematological malignancies.

In conclusion, this report presents a FPD/AML patient with a germline *RUNX1* mutation and an acquired *TET2* mutation leading to clonal haematopoiesis. During a five‐year follow‐up, the haematopoietic clone acquired other genetic alterations including mutations in 18 genes. These subclonal alterations led first to T2‐ALL development, and later to a T/Myeloid phenotype with an AML‐M0 development (Fig. 1).

Clonal haematopoiesis in asymptomatic *RUNX1* carriers under the age of 50 years was already reported 9 suggesting together with our results that the identification of clonal haematopoiesis before leukaemia development in FPD/AML patients could serve as a marker of pre‐leukaemic state. This finding might be helpful in patient care, especially if bone marrow transplantation is considered.

## Conflict of interest disclosure

The authors declare no competing financial interests. The online version of the article contains a data supplement.

## Author contribution

MVT, BH, ADI, AB, MG, DN and DMK performed genetic analysis and analysed mutational data. MVT, BH, VW, PI, ME, AV, FR, BV and RH conceptualized the idea, designed the research and analysed data. FR, PT, CTR and BV provided samples and data. MVT, BH, ADI, ME, AV, BV and RH wrote the manuscript, which was approved by all the authors.

## Supporting information


**Figure S1** Morphology and phenotype of AML‐M0 blasts.
**Figure S2** Comparative genomic hybridization array on T2‐ALL and AML‐M0 blast populations.
**Figure S3** TCRδ and TCRγ rearrangement analysis.

**Table S1** List of primers used for NGS.
**Table S2** Deletions and insertions found in AML‐M0 btasts and/or T2‐ALL blasts by CGH array.
**Table S3** Variants found in AML‐M0 blasts, T2‐ALL blasts and CD34+ cells by NGS.
