# Supplementary material for: Acquired TET2 mutation in one patient with familial platelet disorder with predisposition to AML led to the development of pre‐leukaemic clone resulting in T2‐ALL and AML‐M0
Source: J Cell Mol Med. 2016 Dec 20;21(6):1237–42. doi: 10.1111/jcmm.13051 (PMC5431233; doi:10.1111/jcmm.13051)
Supplement: Supplementary file 1 — Figure S1 Morphology and phenotype of AML‐M0 blasts. Figure S2 Comparative genomic hybridization array on T2‐ALL and AML‐M0 blast populations. Figure S3 TCRδ and TCRγ rearrangement analysis. [file JCMM-21-1237-s001.docx]

***Acquired TET2 mutation in one patient with familial platelet disorder with predisposition to AML led to the development of pre-leukemic clone resulting in T2-ALL and AML-M0***

Manchev Vladimir T.^1,2,*^, Bouzid Hind^1,2,*^, Antony-Debré Iléana^1^, Leite Betty^3^, Meurice Guillaume^4^, Droin Nathalie^1,3^, Prebet Thomas^5^, Costello T. Régis^6^, Vainchenker William^1^, Plo Isabelle^1^, Diop M’boyba^1,4^, Macintyre Elizabeth^7^, Asnafi Vahid^7^, Favier Rémi^1,8^, Baccini Véronique^9^, Raslova Hana^1^.

**Supplemental figures**

**SF1**


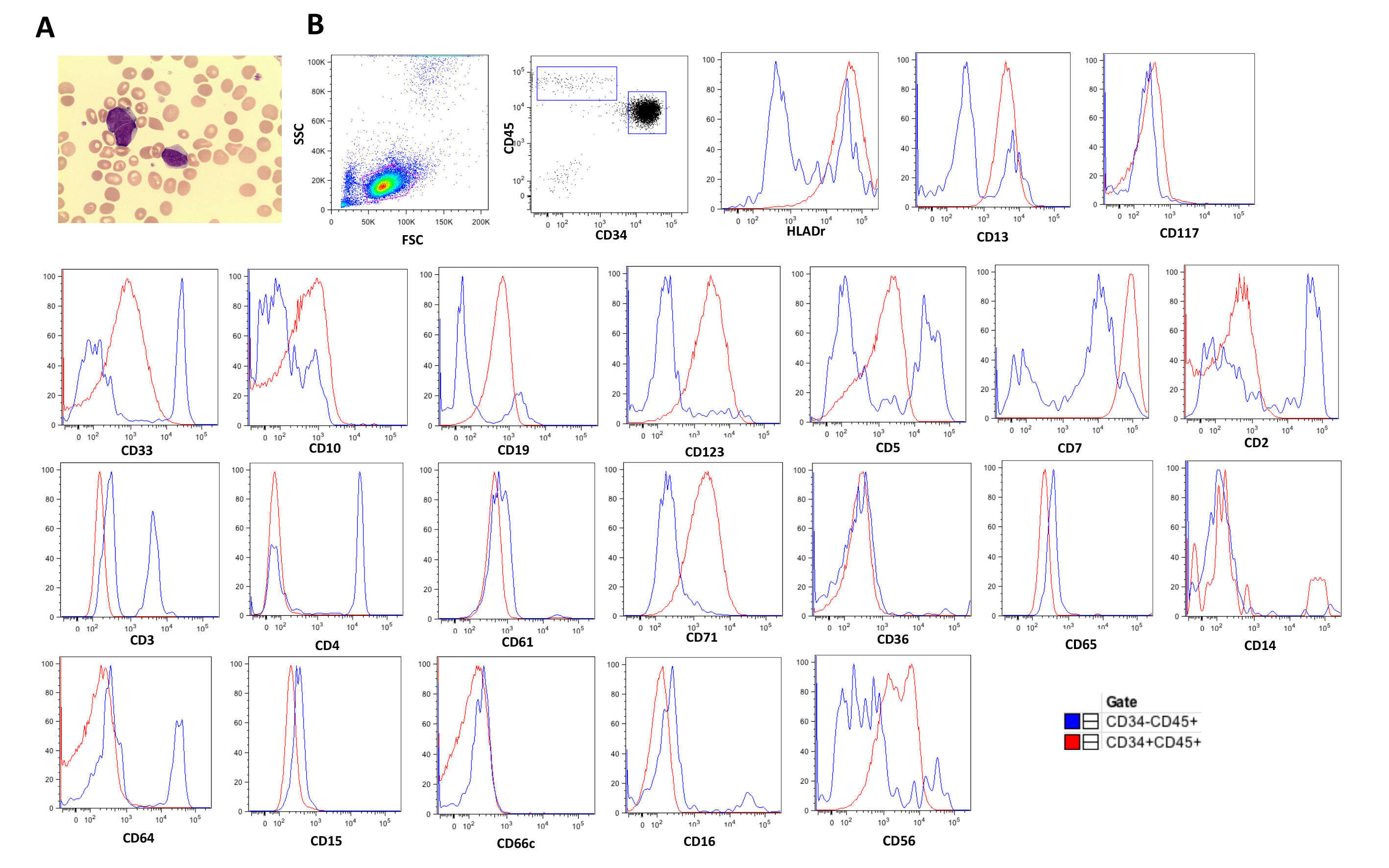


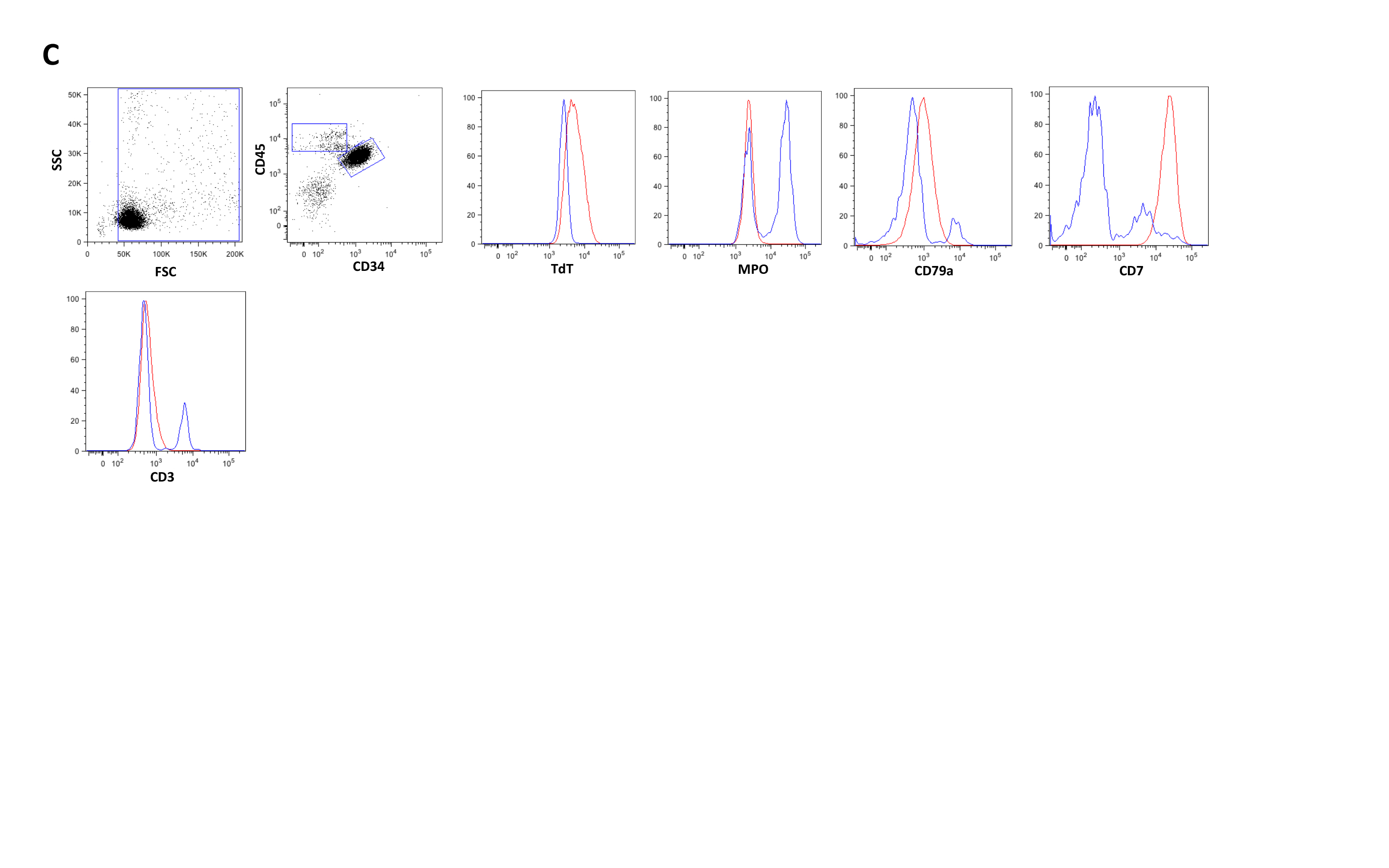


**Supplemental figure 1 :** Morphology and phenotype of AML-M0 blasts.

**A.** May-Grünwald-Giemsa staining of AML-M0 blasts present in peripheral blood. **B, C**: Immunophenotyping of AML-M0 blasts present in peripheral blood, B: surface staining, **C**: cytoplasmic staining.

**SF2**

**
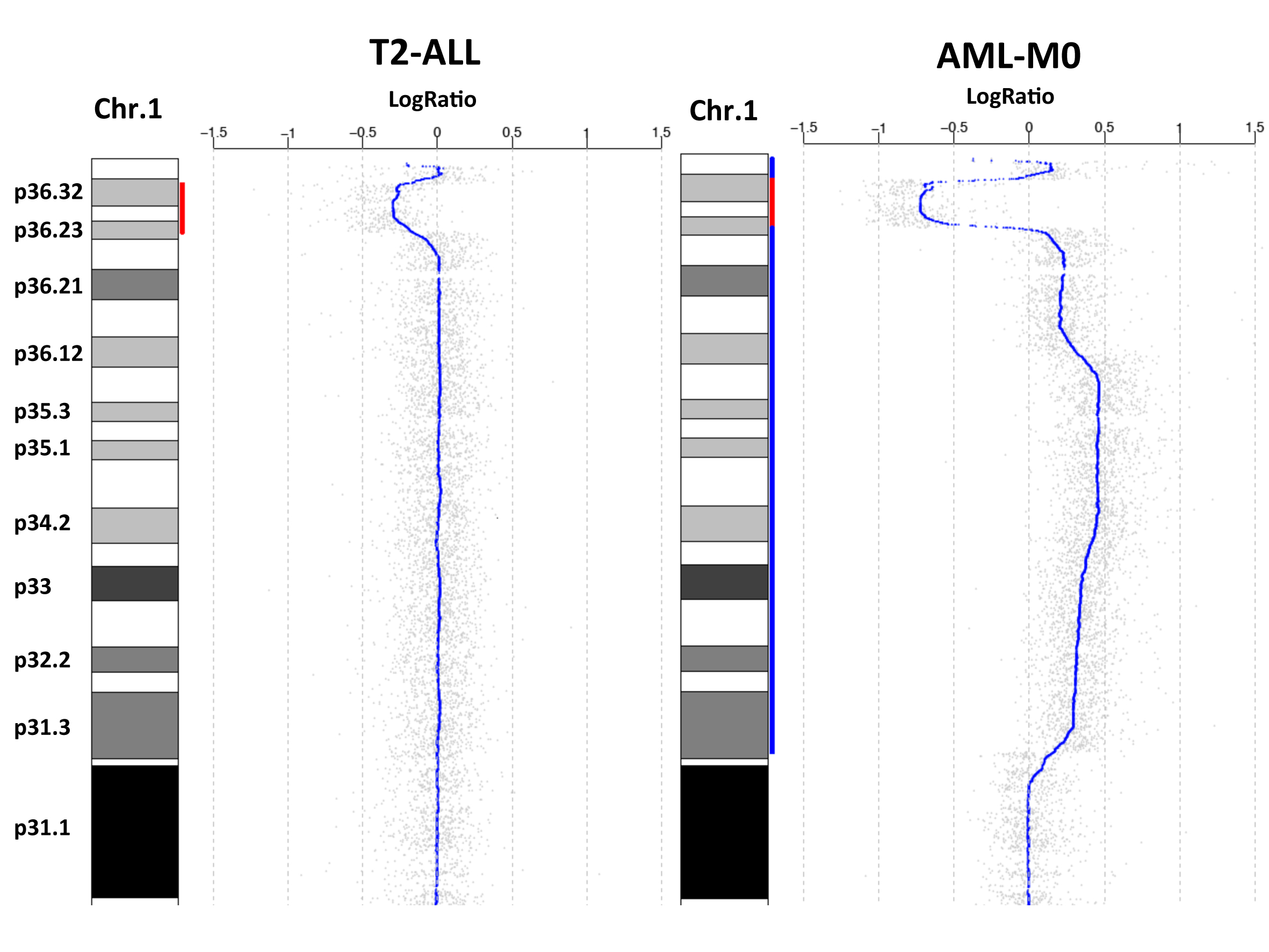
**

**Supplemental figure 2 :** Comparative genomic hybridization array on T2-ALL and AML-M0 blast populations. A focus on 1p36.32-1p31.1region is shown. Red bar indicates deletion and blue bar amplification.

**SF3**

**
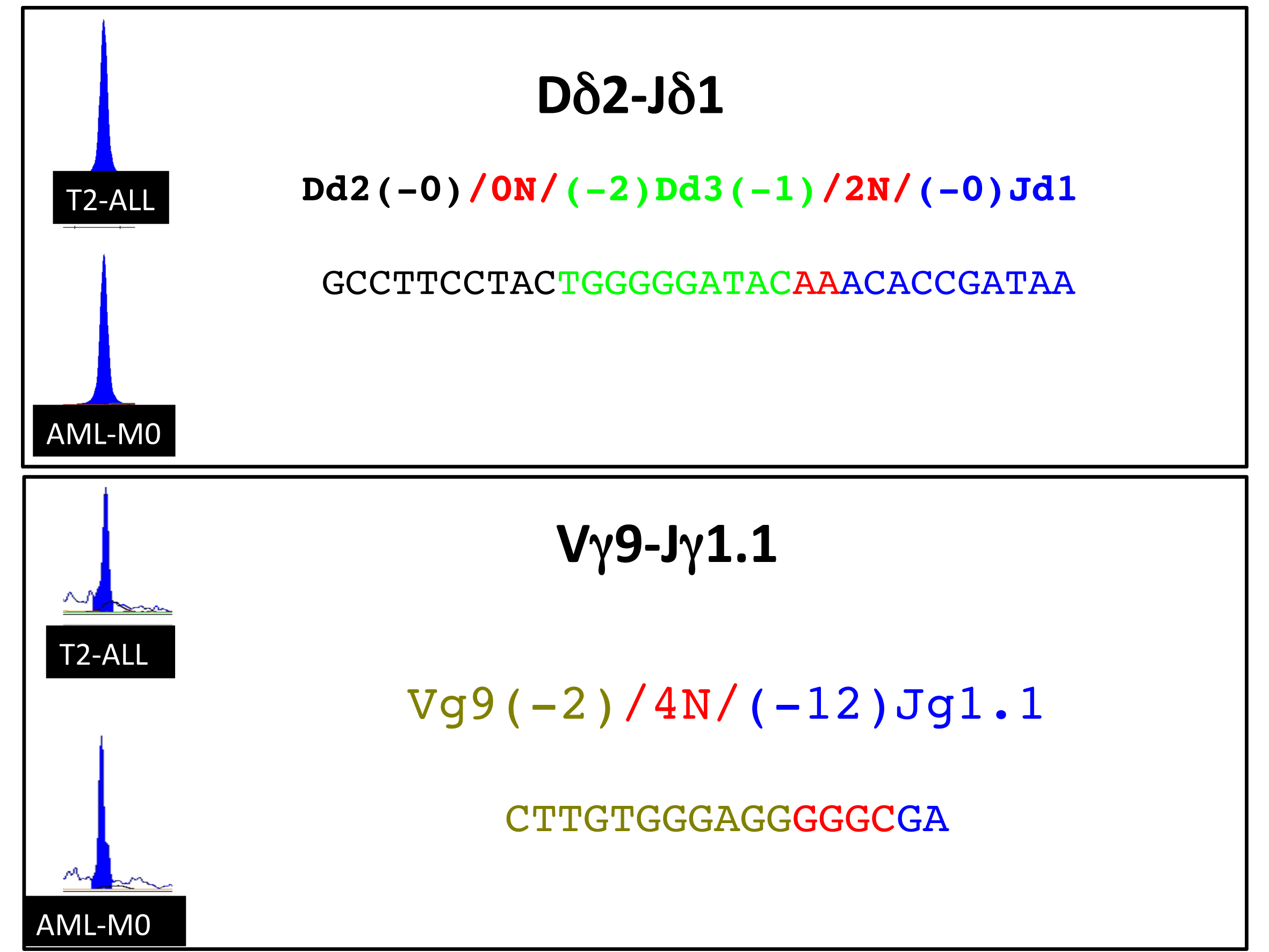
**

**Supplemental figure 3 :** TCRδ and TCRγ rearrangement analysis.

Clonal TCRδ and TCRγ rearrangements by Fluorescent PCR Genescan analysis (left panel) observed in T-ALL and AML-M0 samples. Direct sequencing of rearrangements demonstrated the presence of the same CDR3 sequences, of TCRδ (Dδ2-Jδ1) and TCRγ (Vγ9-Jγ1.1), in both leukemic samples (right panels).
